# COVID Police: Role of Ajman Police During Initial Lockdown

**DOI:** 10.3389/fpsyg.2022.916713

**Published:** 2022-05-19

**Authors:** Osman Sirajeldeen Ahmed, Enaam Yousif, Saeed Ameen Nasef, Alaa Zuhir Al Rawashdeh

**Affiliations:** ^1^Department of Sociology, College of Humanities and Science, Ajman University, Ajman, United Arab Emirates; ^2^Humanities and Social Sciences Research Center (HSSRC), Ajman University, Ajman, United Arab Emirates

**Keywords:** COVID, police, lockdown, responsibility, community

## Introduction

The COVID-19 pandemic has contributed to a “massive global field experience” on how to activate the police force at local levels. This experience has given a new paradigm for police work that has gone beyond the traditional role of the police to shift police in most parts of the world during lockdown to new roles directed toward the public health goal of preventing infection. To emerge a new concept and term called “COVID-19 Police” (Boon-Kuo et al., 2020; Sheptycki, 2020), COVID-19 is also creating a set of unexpected and unprecedented challenges for police departments around the world (Luong, 2020; Zoha, 2021).

The police during the COVID-19 pandemic played an active role in carrying out the mandate to continue to comply with health protocols. Social restrictions are an important phenomenon today for a country (Alharbi et al., 2022), because the violation of social restrictions is difficult to avoid and the role of the police is needed for this policy to succeed. This review aims to clarify the role of the police during the crises of the epidemic, and the expectations of society for the new roles of the COVID police, a case study of the Ajman police in the United Arab Emirates.

Police work has been greatly affected worldwide by the impact of the COVID-19 epidemic; within a short period of time, the police have been assigned new responsibilities with changing priorities and focusing on the enforcement of extraordinary emergency orders. Questions about public expectations and trust in police during the pandemic. The police in most countries of the world have found great difficulties and complications in playing the role of health care on the front lines during COVID-19, taking into consideration the various demographic and economic factors that are different between each country (Alcadipani et al., 2020).

## Research Problem

The research problem focuses on the relationship between the police and society, with a case study of the Ajman Police, from the perspective of the community about the role of police in the period of the spread of COVID-19 during the initial lockdown.

## Research Question

The study seeks to answer the following main question:

Q1. How do community members evaluate the initiatives used by Ajman Police during the COVID-19 crisis?

## Methodology

The electronic questionnaire is the method used to collect data. A five-point Likert scale was used to obtain the respondent's weight (1 = strongly disagree, 2 = disagree, 3 = neutral, 4 = agree, 5 = strongly agree); To calculate the importance or the general estimate for each statement, we calculated the cell length = (the upper limit of the scale - the lower limit of the scale) ÷ the number of degrees of the scale = (1–5) ÷ 5 = 0.8.

Study uses the descriptive survey approach; a survey was conducted of a sample of people of the community in the Emirates of Ajman – total number, 160. A stratified random sample in the Emirate of Ajman had been used, and then a sample size formula by Yamane, n = N/1 + N (e2) was used to calculate the sample size.

## Brief Literature Review

Both Abdoul-Azize and El Gamil (2021) stated that the spread of COVID-19 represents a social crisis that requires the management of social protection policies, especially in the most affected countries. They clarified that social protection becomes a major political tool that serves multiple purposes, whether in countries with high income or low income, and they emphasize that social protection policies are flexible and adaptable tools for social policy makers to face the shocks of epidemiological crises. In this study, we adopted this viewpoint on the grounds that the police are one of the agencies entrusted with implementing social protection policies during the COVID-19 crisis, especially in high-income countries, because the police have recognized civilian roles.

Due to the increasing incidence of various disasters, crisis management has become an important topic for policy makers in many countries, and many researchers mention that epidemics are one of the crises that need to be managed through social protection policies, for example (Rutkowski and Bousquet, 2019). In the same context, Tamer (2004) emphasizes the proactive approach to social protection is widely applied by policy makers in crisis management. This approach aims to reduce social disruption during crises.

Solt (2018) stressed that strategies to mitigate epidemic crises are being implemented by governments, organizations, and institutions and include the implementation of new legal regulations, and the establishment of programs or initiatives to reduce the negative effects of the crisis. The COVID-19 epidemic represents a global threat compared to previous epidemic crises that link specific regional borders.

Luong (2020) provided a systematic model for the role of the police during epidemic in Vietnam. This model relies on the need for the police to approach the people to ensure the effectiveness of the proactive role of the police and the transition from protective measures to preventive measures. It also indicated how police institutions should design and train professional skills and knowledge necessary about transmission of infection for police officers to deal with emergency crises.

Scholars, such as Buil-Gil et al. (2020), focus on other police roles during the lockdown linked to the COVID-19 outbreak by looking at the short-term impact of COVID-19 and lockdown measures on internet-based crime and online fraud in the UK. The study found that cybercrime has increased during the outbreak of the Corona virus, and was.

In another direction, many scholars going to that COVID-19 gave the police an opportunity to build trust with the public and confirm the legitimacy of the police in the societal conscience. Among these scholars is Jones (2020), who explained that it is important for fair procedures between the police and the community that include community safety by giving people the opportunity to express opinions and show transparency and impartiality through quantitative surveys about people's attitudes to the police during the epidemic, and this would provide an important vision about the legitimacy of the police during the pandemic and what effects it may have on post-pandemic times. There are also other scholars on the importance of police legitimacy during crises and epidemics (Tyler and Jackson, 2014; Mazerolle and Wickes, 2015).

Each scholar is interested in studying the psychological effects of police officers due to the COVID-19 epidemic, especially during lockdown. Yuan et al. (2020) argue that police personnel, as first responders to emergencies, are at risk of infection, and also suffer from overtime fatigue and social responsibility; the sudden upheaval of society resulting from the COVID-19 pandemic may affect the mental health of police officers and lead to a variety of psychological problems, such as anxiety and depression.

## Discussion

In this part of the research paper, we will discuss the evaluation of community members in the Emirate of Ajman of the initiatives undertaken by the police to contribute to controlling the spread of the epidemic during the initial closure period, according to the expressions, indicating the opinion of the community about the role of the police, which is shown in the Table 1.

**Table 1:** Ajman Police initiatives in dealing with the (Covid-19) crisis.

**S.D**.	**Items**	**Mean**	**Items**
2	Police and community partnership in controlling the epidemic	4.66	0.623
10	Police confront the expected damage	4.64	0.588
12	Diversity in crisis management	4.63	0.612
6	There have been many activities undertaken by the police to limit the spread of the Corona virus	4.65	0.574
3	Crisis management at the Ajman Police General Command, contributed to the rapid intervention	4.65	0.596
1	Special mission teams played a key role in dealing with the crisis	4.67	0.591
5	Ajman Police have trained cadres to manage health crises	4.65	0.626
7	Community volunteering is one of the methods of managing the Corona virus crisis	4.64	0.638
13	Ajman Police decided to partner with the private sector in controlling the epidemic	4.58	0.649
11	Partnership between the media and Ajman Police contributed to controlling the epidemic	4.63	0.641
8	The management of the epidemic clarified the relationship between medical and police personnel	4.64	0.628
9	Time management is one of the important elements that the police relied on in controlling the epidemic	4.64	0.638
4	The police provided all societal benefits with the obligation to maintain security	4.65	0.675
**Overall**		4.64	0.542

The data of the Table 1 illustrate the public's opinions on the initiatives that have been active through the role played by the Ajman Police in controlling the reality created by COVID-19 during the initial lockdown period. These are as follows:

a. The results reported in Table indicate that most of Community random an average level of community members evaluate the initiatives used by Ajman Police during the Covid-19 crisis with overall mean (4.64) and standard deviation (0.542) for all items.
b. The sample of the study strongly confirms that the presence of the task force of the General Command of Ajman Police was the main factor in implementing the total closure, which was reflected in mitigating the spread of infection, and this achieved the highest arithmetic average (4.67), which falls within the general assessment category (strongly agree), and reached the standard deviation of the statement is 0.591.
c. Partnership initiatives between Ajman Police and the community are among the important points emphasized by the study sample with regard to the COVID police, where the arithmetic mean was 4.66 for this category, which falls within the general assessment category (strongly agree), and the standard deviation of the expression was 0.623.
d. Crisis management at the Ajman Police General Command contributed to the rapid intervention to contain the crisis, one of the important roles emphasized by the study sample with a mean of 4.65, which falls within the general assessment category (strongly agree), and the standard deviation of the expression was 0.596.

## Conclusion

Emergency management in an epidemic situation is a shared social responsibility, and police are an important component of society. Creating a common goal can be difficult when team members are in emergency operations centers. Successful police leaders continue to care for their members during an emergency, remembering that their success as a leader is closely linked to activities coordinating staff for the entire agency to identify and share directions and priorities, and to fill in gaps.

This study showed that the Ajman Police were working in harsh and difficult conditions, especially when combining emergency health tasks with the regular tasks of the police in protecting security. In addition, there are increasing expectations from society for police work during the epidemic, which increased the psychological pressure on the police personnel. Overall, the indicators of the study sample tended toward emphasizing that the Ajman Police had presented community partnership initiatives that contributed to alleviating the infection of the epidemic.

## Author Contributions

All authors listed have made a substantial, direct, and intellectual contribution to the work and approved it for publication.

## Funding

This paper has been supported by the Deanship of Research and Graduate Studies, Ajman University.

## Conflict of Interest

The authors declare that the research was conducted in the absence of any commercial or financial relationships that could be construed as a potential conflict of interest.

## Publisher's Note

All claims expressed in this article are solely those of the authors and do not necessarily represent those of their affiliated organizations, or those of the publisher, the editors and the reviewers. Any product that may be evaluated in this article, or claim that may be made by its manufacturer, is not guaranteed or endorsed by the publisher.
